# Radio Frequency over Fibre Optics Repeater for Mission-Critical Communications: Design, Execution and Test

**DOI:** 10.3390/s22020612

**Published:** 2022-01-13

**Authors:** Răzvan-George Bărtuşică, Mădălin Mihai, Simona Halunga, Octavian Fratu

**Affiliations:** 1 Research and Development Department, The Special Telecommunications Service, 060044 Bucharest, Romania; razvan.bartusica@stsnet.ro (R.-G.B.); madalin.mihai@stsnet.ro (M.M.); 2 Faculty of Electronics, Telecommunications and Information Technology, University Politehnica of Bucharest, 060042 București, Romania

**Keywords:** mission critical, communications, TETRA, public safety, sensors, fiber optics, radio frequency

## Abstract

This paper presents a technical solution that addresses mission-critical communications by extending the radio frequency coverage area using a flexible and scalable architecture. One of the main objectives is to improve both the reaction time and the coordination between mission-critical practitioners, also called public protection and disaster relief users, that operate in emergency scenarios. Mission-critical services such as voice and data should benefit from reliable communication systems that offer high availability, prioritization and flexible architecture. In this paper, we considered Terrestrial Trunked Radio (TETRA), the mobile radio standard used for mission-critical communications, as it has been designed in this respect and is widely used by first responder organizations. Even if RF coverage is designed before network deployment and continuously updated during the lifetime of the technology, some white areas may exist and should be covered by supplementary base stations or repeaters. The model presented in this paper is an optical repeater for TETRA standard that can offer up to 52.6 dB downlink, 65.6 dB uplink gain and up to 3.71 km coverage distance in a radiating cable installation scenario. The design in not limited, as it can be extended to several different mobile radio standards using the same principle. Flexibility and scalability attributes are taken into consideration, as they can build a cost-effective deployment considering both capital and operational expenditures.

## 1. Introduction

Critical communications can be defined as reliable communications between first responders (e.g., police, fire brigade, medical staff, etc.) during mission scenarios. One key element in assuring the success of the mission is the coordination between all the actors, that can be done by sharing critical information in an effective and uniform way. Mission-critical communications rely on operational mobility which provides a uniform service access and level of reliability for users, regardless of the area in which they operate. Another important aspect is the need for high availability of the communication system. A high availability (e.g., at least 99.99% uptime considered for one year reference, or approximately 5 min of downtime per month) represents a good indicator for reliable critical communications.

The Terrestrial Trunked Radio (TETRA) [1,2,3] is a European professional mobile radio standard, especially designed for military communications, for emergency (police, fire, ambulance) services as well as for transport and public safety networks. The system has numerous advantages, of which we can mention the frequency domain used that allows good geographical coverage with a relatively small number of receivers, thus reducing the costs. The communication is end-to-end encrypted and cannot be interrupted even if the mobile terminals are changing site, making it a very robust and reliable system, due also to the modulation and coding techniques used. When the network is not available, the terminals may enter into direct mode where they are directly connected, ensuring the system functionality in difficult emergency situations, in which most of the networks are failing. On the other hand, there are a number of disadvantages, since it necessitates a linear amplifier in order to meet the strict radio specifications and to coexist with other radio technologies, which makes the handsets more expensive. The transmission rates are also relatively low compared to modern application requirements.

However, due to their important advantages, many research teams have tried to develop and extend the existing system, eventually by combining them with different other standards, in order to increase the coverage area, and to improve the overall system, offering a more flexible and scalable architecture, for specific types of operations. In [4], the authors analyze the possibility of integration between TETRA and LTE Technology, in order to benefit from the advantages offered by both system advantages for future 5 and 6G systems, while in [5], the authors present a number of measurement results regarding the co-existence between LTE and TETRA in the same 800 MHz bandwidth in order to optimize their cooperation for critical mission applications. In [6], the authors analyze the threats and vulnerabilities of VoIP and TETRA systems and compare the countermeasures that have to be taken, focusing on confidentiality, integrity and availability, and in [7], several measurement results are presented regarding the GPS accuracy and the Short Data Service (SDS) round-trip-time performances of several real-world scenarios using TETRA in trunked mode. In [8], the authors present several scenarios that enable priority communications (police, border guards, ambulance, fire and rescue) over a commercial mobile network, in order to develop promptly deployable networks for emergency and tactical operations, using prioritization, QoS management, SDN, network slicing, end-to-end security, and spectrum sharing. A heterogeneous solution for ultra-reliable communications is presented in [9], that includes WLAN, LTE and TETRA, enhanced with automated switching between the wireless technologies through Dynamic Link Exchange Protocol (DLEP) in order to ensure a guaranteed availability for voice connection, additionally providing a supplementary data rate for other additional services, such as video. A disaster-resilient three-layered architecture for Public Safety-LTE is presented in [10], that consists of an SDN layer that provides centralized control, a UAV cloudlet layer to facilitate edge computing or to enable the emergency communication link, and a radio access layer. Critical content would be served via TETRA while non-critical content would be offered via broadband communications. 

The importance and applicability of RF repeaters is stated in several pieces of research. The standardized requirements and technical specifications for TETRA systems are described in [1,2,3] while considerations regarding public safety networks and critical communication systems are mentioned in [10,11]. Design and implementation principles for repeaters are found in [12], where several RF-repeater configurations are presented and analyzed based on numerical analysis and field measurements, and in [13] a double channel digital repeater is proposed and evaluated that can work at 2.4 GHz frequency in an indoor and outdoor environment. In [14], the authors present several solutions that can be applied for extending coverage for TETRA networks and the solutions are analyzed based on simulations performed with ICS Telecom. In [15], the authors present an FPGA-based digital repeater that can be customized in order to be used in different scenarios, frequency bandwidths and numbers of channels, and the results obtained based on the FPGA implementation and its corresponding MATLAB emulator are presented. 

The implementation aspects at component level are presented in [16,17,18] and RF over optical fiber (OF) transceiver technical specifications, applicability and limitations are found in [11,19]. A review of radio over fiber (RoF) is presented in [20] with an evaluation of its current use, potential and challenges for future applications. Newer research regarding radio over OF are also presented in [21], where an optimized digital RoF system is presented for the transmission of LTE 20 MHz signals with 64 QAM modulation; the analytical model developed is presented validated experimentally for 30 km of fiber length. In [22], the authors present the design of an optimal RoF system and validate it based on OptiSystem software simulation.

In [23], several radio planning solutions for the TETRA to LTE migration for public protection and disaster relief networks are offered, and their functionality is tested both by simulation and measurements. Testing and validation principles are also presented in [23,24,25,26]. In [27], the authors extended the coverage of the narrowband technologies (TETRA, P-25 and Tetrapol) to extend their coverage areas for mission-critical applications using digital mobile radio, and tested their system for the Attica region. The authors of [28] studied different combinations and configurations of existing communication technologies (narrowband push-to-talk, LTE, 5G new radio and WiFi) to provide a mission-critical system that offers reliable services and extended coverage areas.

The novelty in the study is highlighted on three layers: the operational aspect which allows a fast and flexible installation depending on the scenario requirements, the technical aspect that offers the benefit of tailoring the technical solution regarding the signal pick-up, radio coverage and service quality assurance, and a resilient approach both system monitoring and functional control. 

The paper is organized as follows: in Section 2, the TETRA system characteristics are briefly defined; Section 3 presents the use case considerations for a sensor-based approach and several design considerations, Section 4 presents different RF pick-up modes, while Section 5 details the optical repeater internal architecture and gain considerations. In Section 6, link budget values are estimated, while Section 7 describes the remote monitoring and control solution for an optical repeater, as a web-based application for control rooms such as network operation centers that can be easily extended to a solution for an entire network of repeaters. Test and validation measurements are described in Section 8, considering gain measurement, various services testing, modulation quality measurements and an automated testing procedure from service side. Section 9 is reserved for result synthesis and discussion. Conclusions and future work are presented in Section 10.

## 2. General Characteristics of TETRA Communication Systems

The TETRA radio communication system is designed for mission-critical applications, considering a high availability mode of operating: communication inside network coverage (trunked mode operation—TMO) and off-network (direct mode operation—DMO). Direct mode can be further operated either in device to device or local group communications, allowing a high level of cooperation between users. On the other hand, TMO allows intra-agency and inter-agency communications by group communication functionality under network coverage. Narrow-band voice and data services are provided, with a maximum 300 ms call setup time. Cooperation is supported by the dispatch feature adding a layer of control and coordination. As a mission-critical communication system, prioritization for both network access and data traffic is possible. The system allows security mechanisms such as authorization, authentication and encryption (including end-to-end encryption) while messaging and IP communication are performed by short data and packet data services. The access method TDMA, with four timeslots per channel, one channel having 25 kHz bandwidth with 28.8 kbps data rate (per channel). The modulation scheme is $\frac{\pi }{4}DQPSK$ digital modulation [1,2,3].

### 2.1. Coverage Considerations

Depending on coverage requirements several repeater models exist on the market [14,29,30,31,32,33,34,35,36]:

- Reduced coverage area repeaters, which can offer around +20 dBm output RF power. These repeaters are well suited for small and open-area offices, control rooms, police and fire brigade office stations. These models are compact, low cost and have the advantage of being easy to deploy;
- Medium coverage area repeaters, used in building deployments, underground metro stations, parking garages, short tunnels and even open area. The output power of such models can be up to 1 Watt (+30 dBm);
- High coverage area repeaters. This category addresses the big scale coverage issues being the solution for remote tourist attractions, small villages and cities, multistore buildings, large underground infrastructures. It is expected to provide a high output RF power, usually up to +40 dBm. These models involve a complex design, proper RF planning and tests after deployment, in order to update and optimize existing network coverage.

### 2.2. Signal Pick-Up Considerations

Considering the signal pick-up model, two types of repeaters can be defined [33,36]:

- Off-air pick-up repeaters. The link to the base station (BS) is usually made through directional high-gain antennas in order to compensate for free space loss. They represent a suitable solution for long distance to BS scenarios;
- Direct connection to BS repeaters. The link to BS is made through a coupler. Supplementary RF attenuation should be taken into consideration in order to limit the power into the BS/repeater. This measure is mandatory for avoiding the overload state of active components.

### 2.3. Selectivity Considerations 

Selectivity criterion defines another two types of repeaters:

- Band-selective repeaters, which retransmit the whole uplink and downlink bands (e.g., Tetra standard has a 5 MHz bandwidth for both UL and DL). This solution can be used in scenarios where a high capacity is needed, users being able to register and communicate on different BSs. An important drawback, unfortunately, should be mentioned: the performance of amplifiers over a large bandwidth;
- Channel-selective repeaters. The retransmission is performed over a limited number of control and traffic channels, with a low bandwidth (e.g., 25 kHz/channel for TETRA standard). Better performance over bandwidth is obtained, capacity can be controlled by extending the number of channels up to eight (depending on the BS configuration) but this solution consists of a more complex design. An important shortcoming should be stated: in the case of BS frequency reallocation/replanning, the channel selective repeater must be reconfigured in order to assure continuous service provisioning.

## 3. Use Case Applications and Design Consideration for Network Approach

**Figure 1:**
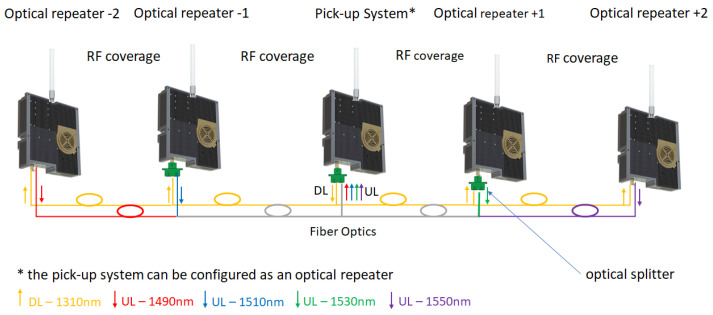
High level architecture for a cluster of 5 optical repeaters.

Mission-critical communications, such as voice and data, successfully address public safety and emergency response aspects. As voice communications are well covered by different modes of operation, centralized coordination and grouping features, messaging and data services are also important issues that should be taken into consideration in mission-critical communications. Developing sensor applications in public safety and emergency-response solutions can be of great benefit as TETRA is still one of the most reliable wireless networks with respect to mission-critical communications. Integration of different types of sensors in such applications can be performed by messaging and packet data features correlated to TETRA specifications. An essential feature related to critical communication is prioritization and pre-emption which allows prioritized access to network resources. One example is Short Data Service (SDS), described in Section 8.4 for one of our test and validation procedures. Sensor data can be encapsulated in SDSs, as shown in Figure 1 and sent to a control room endpoint or application over TETRA infrastructure. This method benefits from the prioritization due to the SDS best service approach: SDSs are sent on the first slot of the main control channel (MCCH), in contrast to other wireless communication systems that may be using the best-effort approach when dealing with messaging and data. Another method of transporting sensor data is by packet data service (PD), which can establish UDP and TCP communication on TETRA’s traffic channels (TCH).

To conclude, the designed optical repeater along with TETRA mission-critical communications is well suited in applications where public safety and emergency sensors must benefit from network coverage, prioritization and reliable communication.

This work addresses the practical aspects of building an optical repeater, as an experimental research model transitioning to a real-life operational prototype solution that can be integrated in a cluster or network, as shown below. The RF over fiber converter used for TETRA coverage extension has a wide RF bandwidth (from 30 MHz to 2.7 GHz) and is equipped with an internal RF low noise amplifier of 20 dB. It converts the RF signal into an optical signal using a linear distributed feedback (DFB) laser (TxFO) and re-converts the optical signal into RF by a linear photodiode (RxFO). The optical transmitters have different wavelengths in order to reduce the interference for the uplink path (although TETRA uses TDM frames and there is a floor control implemented at BS side, a possible scenario is that the repeater can extend several BSs so the separation is both ways: time and wavelength).

The architecture design for an optical repeater network comprises of one RF pick-up system and one or more optical repeaters distributed either in star or daisy-chain topology. The link between the pick-up system and its optical repeater(s) consists of one or two fiber optics (one in the case of WDM/CWDM and two for separate optical Tx and Rx daisy-chains). Depending on the number of repeaters, one or more wavelengths can be selected for use:

- *P2P architecture—*uses one fiber in wavelength division multiplexing (WDM) mode (e.g., downlink 1310 nm and uplink 1550 nm). This architecture is based on a pick-up system and one optical repeater. It covers the scenario where a single remote area should be covered;
- *Clustered architecture—*uses several repeaters (in our example five repeaters), therefore two fibers are necessary, one for the downlink path (e.g., 1310 nm) and one for each optical repeater’s corresponding uplink path (e.g., 1490 nm, 1510 nm, 1530 nm and 1550 nm). The reason for two separate paths is to limit optical receiver interference in a distributed architecture, as shown in Figure 1. In this application a five-repeater scenario was considered due to an operational requirement: a central repeater and two by two adjacent repeaters. This architecture has an equal amount of attenuation for each side (two optical splitters and a relatively equal fiber length for each side), even though this would not necessarily be a limitation.

The maximum number of optical repeaters is limited by the optical attenuation between the pick-up system transceiver and the last transceiver from design. The limitation is due to low dynamic range on the optical transceiver side, usually 15 dBo (optical loss dB) mentioned in the provider’s technical specifications. As shown in Section 8, tests should be made prior installation in order to validate proper functioning at higher attenuation values. Pushing the attenuation limit should be backed up by an error control mechanism implemented in the monitoring and control solution which evaluates at least the optical power received from the corresponding RF over OF convertor.

Besides network side error control, an automated quantitative test should be performed from the service side (e.g., the percentage of successful messages received related to messages transmitted). This procedure involves configuring a mobile station to transmit periodic messages to another mobile station or application. Each message should contain at least the channel frequency, received signal strength indication and a message counter.

An optical repeater for mobile communications uses transceivers from the RF to optical domain in order to transport the signal at the remote site where the reverse conversion is performed. At least a pair of RF over OF transceivers are used, for example in a point-to-point deployment. The maximum distance between the transceivers can be estimated as:

(1) $$d_{km}=\frac{DNR_{dB}}{att_{dB/km}},$$

where $DNR_{dB}$ is the dynamic range of transceivers considered for the optical link and $att_{dB/km}$ is the optical link attenuation including the loss inserted by fiber, patch-panels, connectors, optical distribution frames, splitters, etc. The time delay estimations should also be considered due to the TETRA frame structure which should not exceed the maximum cell radius of TETRA terrestrial cell of 58 km or 193 µs round-trip time, according to [1,2].

(2) $$t_{58km}\geq t_{prop}+t_{rep}+t_{proc},$$

where $t_{58km}$ is the time delay corresponding to maximum theoretical cell radius, $t_{prop}$ is the propagation delay, $t_{rep}$ is the extended coverage delay and $t_{proc}$ is repeater processing delay. Different operating scenarios for this design are possible, such as buildings, tunnels and open areas with insufficient or no radio coverage, but also scenarios where there is a need for increased traffic capacity.

## 4. The Pick-Up System

**Figure 2:**
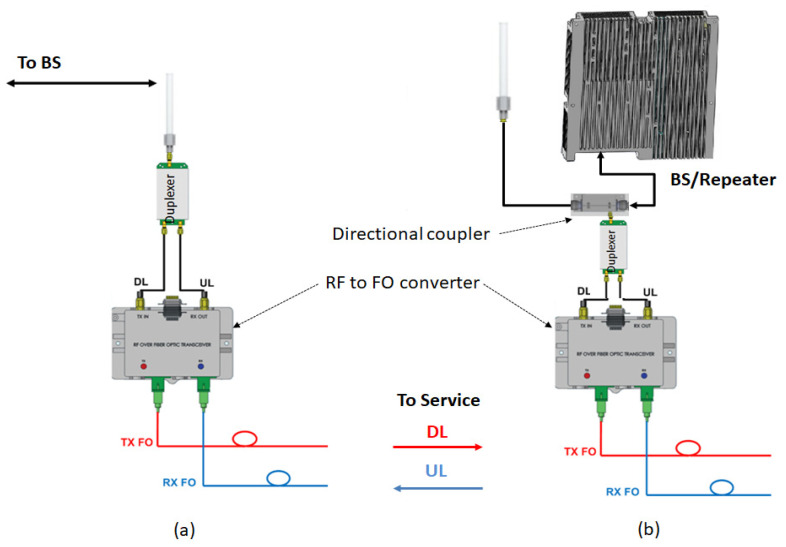
Pick-up modes: (**a**) by antenna, (**b**) direct connection to BS or repeater.

**Figure 3:**
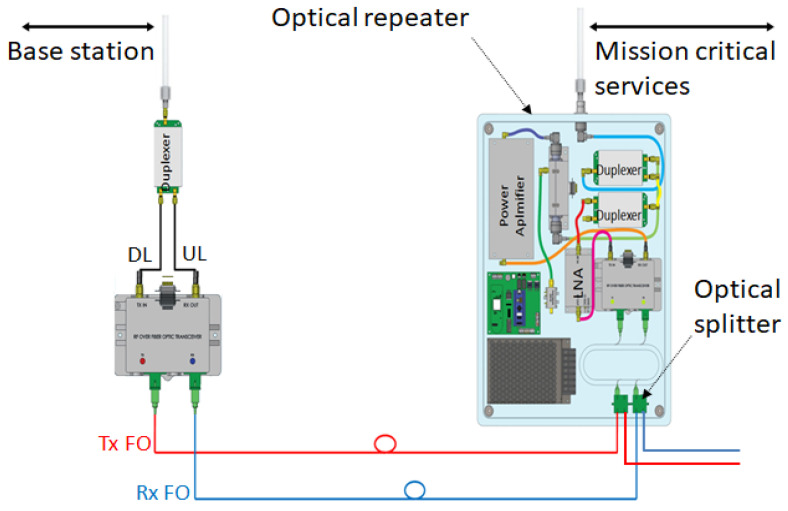
Left—simple pick-up system, right—optical repeater.

The pick-up system represents the RF coupling point to the TETRA network that also converts RF to OF and distributes the signal to optical repeater(s). Several approaches are possible [32,33,36] such as:

- Pick-up mode—that can be designed as off-air, by using antennas, as presented in Figure 2a or direct connection to a BS/repeater through RF couplers, as shown in Figure 2b. Depending on operational requirements, the pick-up system ranges from a simple design, see Figure 2a,b, to a more complex one which includes several active stages, RF and OF splitters and service provisioning in the pick-up area, see Figure 1;
- Service distribution—RF coverage provided only to remote sites, as shown in Figure 3, or both in pick-up and remote sites in a cluster deployment, as depicted in Figure 1. If more than one repeater is needed, optical splitters are used for signal distribution. In this case, the overall optical attenuation should be recalculated in order to ensure the restrictions regarding optical link loss. In this respect, unbalanced optical splitters can be used, with different attenuation ratios (e.g., 25/75, 20/80) or balanced (50/50).

Estimations on overall optical link loss, limited to 15 dBo by the technical specifications, concluded that a maximum number of four optical repeaters can be used, distributed two by two, adjacent to pick-up system, which can also be configured to provide local radio coverage. In total, we estimated a maximum of five deployment sites for mission-critical communication per one pick-up system, distributed in a daisy-chain topology.

## 5. The Optical Repeater

The mode of operation is based on conversion from OF to RF for the downlink path and RF to OF for uplink path, as shown in Figure 3. RF signal amplification is performed as follows:

- DL path—20 dB at pick-up system’s RF over OF converter and 46 dB corresponding to repeater’s power amplifier;
- UL path—20 dB at repeater’s RF over OF converter and 30 dB corresponding to repeater’s low noise amplifier (LNA);

The components were integrated in a custom-built case with the main goal to provide the optimal RF gain and ingress protection, and are listed as follows:

- Balanced optical splitter 50/50 for daisy-chain topology;
- RF over OF converter with integrated LNA of 20 dB on RF Rx path;
- Power amplifier (PA) of 46 dB gain and 37 dBm power output;
- Directional coupler (DC) of 30 dB used for monitoring the PA;
- Power detector used for monitoring the PA;
- Three port circulator terminated with 50 Ω load used for PA protection (playing the role of isolator);
- Two cascaded duplexers (DUP) used for high isolation between UL and DL (>130 dB);
- Control board used as a power supply, monitoring and control solution for active components;
- General power supply;
- IP68 external mounted case fan.

The power target for optical repeater output is desired to be as high as possible which leads to link budget calculations and low loss control for RF and OF paths. 

Taking into account the worst-case deployment scenario, for a daisy-chain fiber optics topology, repeater gain estimations are presented in Table 1.

## 6. Link Budget

The calculation was considered from the perspective of antenna and radiating cable as possible installation scenarios. As prerequisites we consider a system that operates at 400 MHz, a base station (BS) with a power of +43 dBm, a pick-up system placed at *d* = 1 km from the BS, the free space loss (FSL) is estimated at −84.5 dB and the transmitter and receiver have the antenna gain G*_ant_* = 10.5 dBi. The received signal power at pick-up system was estimated to:

(3) $$RSSI=BS_{pwr}-FSL_{1km}+G_{ant}=-31\;\mathrm{dBm}$$

where

(4) $$FSL=20\cdot \log_{10}\left(d_{km}\right)+20\cdot \log_{10}\left(f_{MHz}\right)+32.44$$

The DL power budget at the output of the repeater is:

(5) $$P_{out}=-31\;\mathrm{dBm}+52.6\;\mathrm{dB}=+21.6\;\mathrm{dBm}$$

In order to estimate the coverage distance, we considered a minimum received signal level for a TETRA mobile station of −100 dBm. A radiating cable installation scenario was taken into consideration. The cable performance at 400 MHz is a longitudinal loss ($L_{loss}$) of 18.2 dB over 1 km and a coupling loss ($C_{loss}$) of 54 dB. The maximum distance is given, then, by:

(6) $$-100\;\mathrm{dBm}=P_{out}-d_{rep}\times L_{loss}-C_{loss}$$

which yields a distance for the repeater of $d_{rep}=3.71\;\mathrm{km}$.

On the UL path, the mobile station has an output power ($MS_{pwr}$) of +30 dBm. The signal level measured by the base station in this scenario is:

(7) $$BS_{meas}=MS_{pwr}-d_{rep}\times L_{loss}-C_{loss}+G_{UL}+G_{ant}-FSL_{1km}$$

which gives a total value of **−99.9 dBm**. 

The optical fiber path will introduce additional loss (approximatively 0.8 dB/km RF equivalent) which will reduce the estimated distance accordingly. For a 3.71 km fiber optic path, the corresponding additional attenuation can be estimated at 3 dB.

## 7. Monitoring and Control Design

The state of the system and control functions are one of the most important elements in designing a mission-critical communication platform. Information regarding the performance of both the platform and individual elements should be continuously sent to the control room for monitoring and decision making.

Connection oriented solutions should also be taken into consideration, such as error control mechanisms, data frame counters and uptime for each controller, confirmations, etc. Redundant communication links, other than the ones monitored, should be implemented and used for this process. The control board should integrate at least two communication links (e.g., an ethernet controller and an GPRS/LTE modem). In this respect, we designed the monitoring and control platform as an IoT solution, based on MQTT standard which uses the publish/subscribe pattern. The board accumulates the sensor data, structures it into JavaScript Object Notation (JSON) format and publishes to the MQTT broker.

The control room is a client that subscribes to the published messages, logs the values (e.g., a NoSQL database) and displays it in a web-based dashboard with graphical and numerical representation.

**Figure 4:**
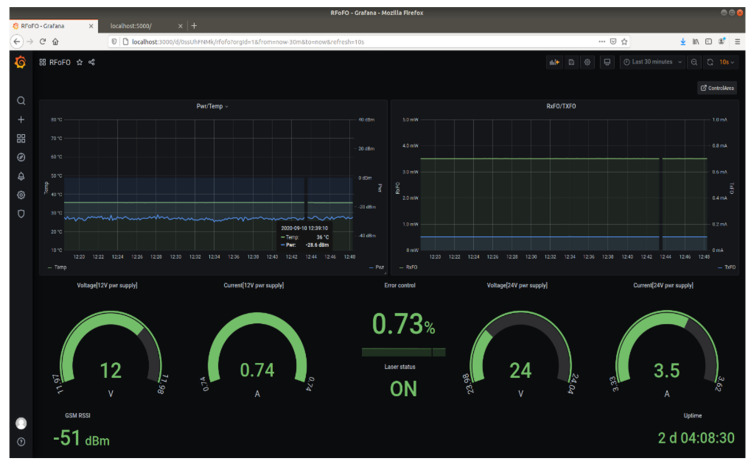
Monitoring dashboard.

The monitoring dashboard, presented in Figure 4, is built as a web application showing a collection of parameters that should be considered for proper operation. The data is periodically displayed and evaluated by querying a database of time-stamped recorded data created for long-term storage.

The monitored parameters are defined as follows:

- *Pwr/Temp graph*: The PA output power (green curve) indicated by the power detector. The value is corrected by 30 dB which is the coupling value of the directional coupler connected to the output of the PA. The temperature (blue curve) is measured by the temperature sensor mounted on the case of the PA. An alert is triggered if the temperature rises above 50 °C and an alarm and shutdown is enforced at 60 °C.
- *RxFO/TxFO*: These are the optical converter parameters that indicate the received optical power (RxFO) and the bias current (TxFO) for a linear mode of operation. In a daisy-chain architecture these parameters indicate not only the local status but also the status of adjacent converters. In the event of a malfunction, the root cause can be determined by assessing RxFO and TxFO.
- *Voltage and current gauges*: The power supply status and consumption for active components. Alarms are also configured for those parameters.
- *Error control*: The controller reports errors in case of communications issues. For example, if data packets are lost or the endpoint is unavailable, the error counter is incremented. An error percentage can be calculated considering the number of errors detected in uptime intervals. An example regarding communication errors is described in Section 8—Testing and validation.
- *Laser status*: This parameter is Boolean and indicates the state of the converter’s laser.
- *GSM RSSI*: The communication link between each repeater and the broker can be established either on an ethernet interface and routed through a private network or the Internet, or it can use public land mobile networks with GSM access. The received signal strength indicator (RSSI) is a measure of the local GSM signal quality.
- *Uptime*: Offers information regarding the repeater’s functioning interval. It is used for error evaluation and indicates the time interval between successive reset actions.

In the event of a malfunction, an alarm feature has also been provided. The persons in charge of monitoring should be promptly notified regarding any issues that may impact the services allowing them to take appropriate actions. In this respect, the alarm triggers have been defined considering the level thresholds and data connectivity status. As alarm events are highlighted, the user can access the control page from the monitoring dashboard interface.

**Figure 5:**
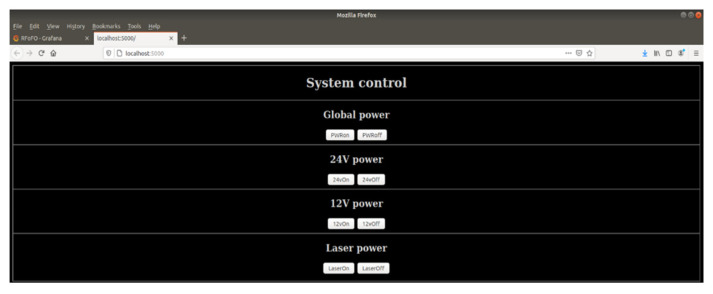
Control dashboard.

In order to remotely perform basic functional operations, access to the control web page of the corresponding repeater is granted directly from the monitoring dashboard. The control mechanism is also web-based, allowing back-end actions triggered by HTML POST methods. The control web page is shown in Figure 5. The user is able to perform basic operations such as:

- Resetting the repeater (global power);
- Turning on and off the power amplifier’s power supply (24 V power);
- Control the 12 V power supply for low noise amplifier, RF to OF converter, power detector (12 V power);
- Turning on and off the laser (laser power).

The entire monitoring and control solution is provided as a virtualized solution and optimized as user-friendly. A long term performance evaluation can also be performed, either by live monitoring the dashboard or offline, by analyzing the data stored in the database with custom-developed tools. The method of integrating the short-term and long-term performance can be a useful indicator for the sustainability of the project.

## 8. Testing and Validation

The testing and validation methodology was inspired by *DRIVER+ Trial Guidance Methodology Handbook TGM* [28] as it provides a comprehensive way for the preparation, execution and evaluation of the project. TGM’s stepped approach offered a better understanding of:

- Structuring the processes;
- Context and scenario;
- Gaps and risks involved in our project, starting from the blueprint and ending with real operational environment;
- Defining the key performance indicators used for evaluation as measurable parameters (e.g., errors over time and number of data frames or the number of successful messages received compared to the message counter at the sender’s side);
- The result analysis process, including conclusion drawing and decision making.

Several tests have been conducted prior to validating the entire system. 

### 8.1. Radiating Cable Installation Scenario

**Figure 6:**
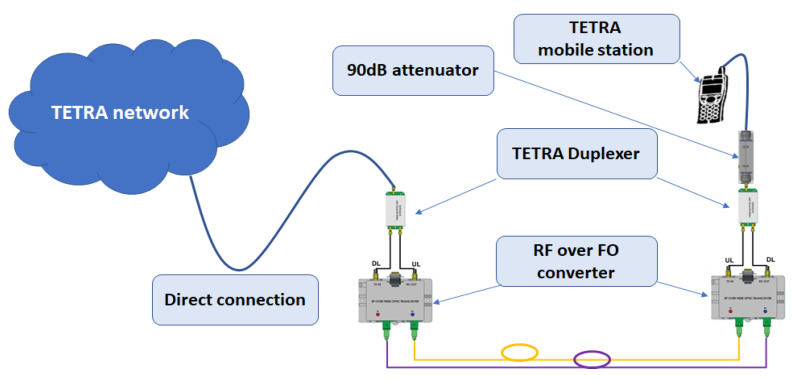
Testbed for 2 km radiating cable scenario.

This scenario was the first validation test which consisted of simulating the path loss for a 2 km path, as described in Section 6 and Equations (4)–(8). Taking into consideration 90 dB attenuation (54 dB coupling loss and 36 dB longitudinal loss) and a supplementary 4 dB duplexer attenuation, we tested the voice service, having the mobile station directly connected to the attenuator, as shown in Figure 6.

For a DL pick-up level of −20 dBm, we measured −98 dBm (note that the RF over OF converters are equipped with a 20 dB low noise amplifier on the RF receiving path) on our mobile station, with the voice service successfully validated in group and private calls.

### 8.2. Optical Fiber Link Maximum Attenuation

**Figure 7:**
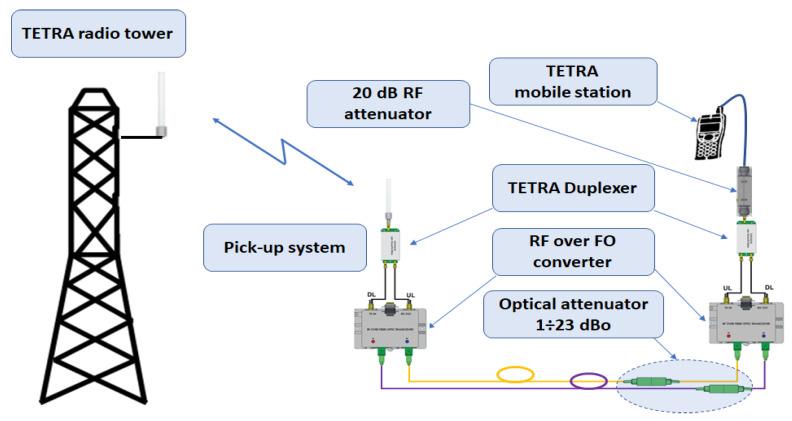
Testbed for determination of RF over OF converter sensitivity threshold.

The second test is performed on the setup presented in Figure 7 and provided several interesting results. Even if the manufacturer of RF over OF converters provided a maximum 15 dBo attenuation between two converters, we tested and concluded that the services can be available at 23 dBo optical attenuation, where 1 dBo is considered as 1 dB optical loss. The dBo notation should remove any confusion between optical attenuation and RF attenuation considering that 1 dBo (1 dB optical loss) has a corresponding 2 dB of RF loss due to the opto-electric conversion. This result can be a good opportunity for inserting two more optical receivers increasing the number of optical receivers and covering areas from 5 to 7 (see Figure 1).

The sensitivity threshold was determined by increasing the optical attenuation by a step of 1 dBo up to the limit where the mobile station disconnected from the network. The test was performed four times in order validate the 23 dBo limit. The difference of 8 dBo offers the possibility to add another optical repeater on both sides of the branches. The 20 dB RF attenuator which was inserted in order to protect the RF over OF converter’s LNA from overload can also be substituted with a supplementary 10 dBo optical attenuation, resulting in a theoretical 33 dBo maximum attenuation for the optical link. Note that a high attenuation on optical link, produces a low RF output power on the service side, which in some scenarios can become an impractical approach for a wide coverage area deployment, but still practical in small areas such as small or open-area offices. The reason we should leverage the capabilities of this solution and increase the number of optical repeaters in the deployment is based on cost efficiency and can be justified as follows: suppose the installation scenario is by direct coupling to a BS. RF pick-up power is high enough to insert another optical repeater at the end of both branches and benefit from supplementary TETRA coverage in an underground parking lot and a small building. With proper link budget calculations, on-site measurements and optical and RF power monitoring, service interruption will be minimized to an acceptable risk. Another example may be an underground metro network. In this deployment, the pick-up may also be performed by direct coupling to one or many BSs, depending on the metro underground architecture. The goal is to invest in a minimum number of BSs and a maximum number of optical repeaters that will be installed in underground stations. 

### 8.3. Measurement of the Effective Gain

**Figure 8:**
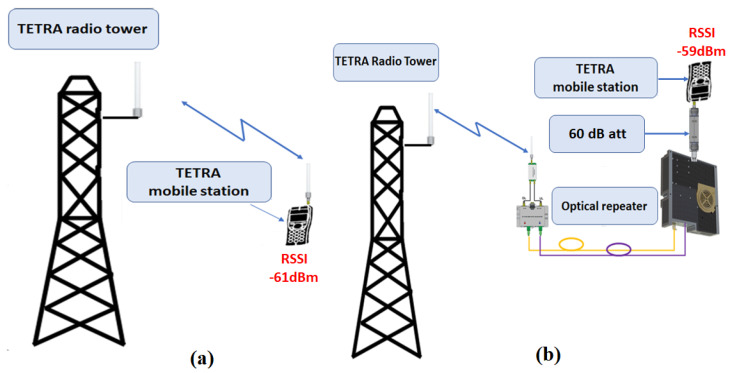
Gain measurement, mobile station readout: (**a**) RSSI measured at pick-up antenna; (**b**) RSSI measured at output port of the repeater with 60 dB attenuation.

The effective gain of the repeater for the DL path was evaluated by measuring the RSSI indicated by the mobile station and applying a substitution method. The reference measurement of the RSSI was taken by connecting the pick-up antenna directly to the mobile station, as shown in Figure 8a, resulting in an RSSI of −61 dBm. Then, by using the testbed according to Figure 8b, we obtained −59 dBm, which concluded an effective gain of 65 dB, according to Equation (8). We took all necessary measures that the same BS was serving the mobile station in both scenarios.

The measured DL gain is:

(8) $$G_{rep}=P_{rep}-P_{ant}+att_{rep}+DUP+att_{sup}=65\mathrm{dB}$$

where $P_{rep}$ is the power measured by the mobile station at the repeater output, $P_{ant}$ is the power measured by the mobile station at pick-up antenna, $att_{rep}$ is the attenuation inserted at output port of the repeater DUP is the attenuation inserted by the duplexer (typ. 2 dB) and $att_{sup}$ is the supplementary attenuation (at least 1 dB) inserted by RF cables, OF patch and connections.

**Figure 9:**
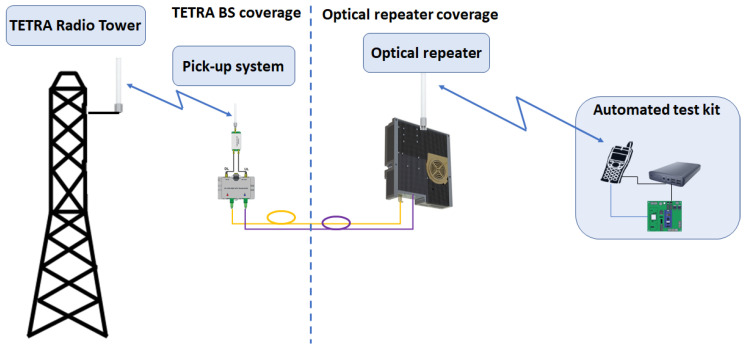
Automated test setup.

**Figure 10:**
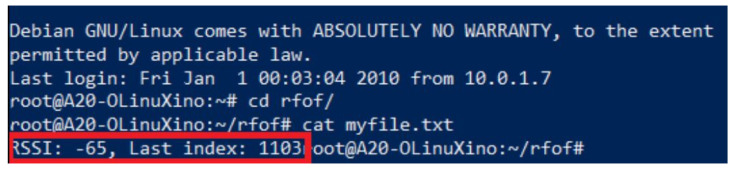
The content of log file.

Further testing was executed by configuring a testbed according to Figure 3 and Figure 9. The procedure was performed in a pass/fail manner and it consisted of:

- Measurement of DL gain over the carrier, Figure 8 and Equation (8);
- Power and temperature over time, Figure 4;
- Measurement of optical received power;
- Group call in trunked mode of operation (TMO);
- Private call over TMO and PSTN;
- Modulation quality, Figure 10 and Table 2;
- Automated messaging in a 24-hour trial, Figure 9;
- Control settings.

### 8.4. Service-Side Testing

This test was performed in order to obtain an indicator regarding the continuity of providing services over a given period of time. For voice services, several tests were successfully performed, but voice service testing is impractical over a large interval (e.g., 24 hours of continuous testing). Thus, an automated testing procedure is needed for this case. In this respect, we developed a system comprising a mobile station, a power-bank, and a single board computer (SBC), as described in Figure 9.

By periodically sending status messages from the automated test kit, we were able to make both quantitative and qualitative evaluations.

- We used Python scripting in order to access the serial interface of the mobile station and send AT commands to extract the RSSI and other parameters reported by the mobile station, according to Figure 10. The above information alongside a message counter was coded in a Short Data Service (SDS) message and transmitted at intervals of one minute to a receiving party that counted received messages, as shown in Figure 10.

After a session of 18 hours, we successfully received 98% of transmitted messages. SDS has also several other important implementations such as telemetry, database query and automatic resource location applications.

- The code presented in Algorithm 1 consists of a loop that executes a series of procedures. By creating a serial communication object, we were able to connect to the mobile station, send AT commands and receive responses. The data received was parsed using a lookup table method and the measured RSSI was logged. Next, we constructed the SDS message that the mobile station should send over the TETRA network using the previously RSSI value and a message counter. After sending the SDS message, we logged the data consisting of current message index and RSSI in a text file stored in the memory of our SBC that we also used for controlling the mobile station.
- During the interval that elapsed between the initiation and the end of our automated test, we successfully received 1085 out of 1103 messages. Although further improvement of the Python code related to timing and serial control could have improved the 98% success, we considered this test as a PASS.

**Algorithm 1.** Serial connection, RSSI request, message construction and logfile update.
**Input:** serial object **while** continuous loop until exception or program interruption **do** Serial connection to mobile station RRSI request Response read and parse result in variable (result) Close serial connection **if** result is not valid **then** Exception handling **else** (program continuation)   Conversion of result according to a RSSI lookup table   Increment the message counter (msg_count++)   SDS message construction (contains result and msg_count)   Serial connection to mobile station   Send SDS message to the destination   Close serial connection   Write content of SDS message in local logfile **end**
**end**



Content of the log file consisting of the last measured RSSI and the message count is marked in Figure 10.

The use of a programable automatic test kit as an acquisition system represents a practical and efficient method not only for service side testing, but also for different applications such as: environmental monitoring (e.g., temperature, humidity, gas leakage, etc.), conditional triggering (access control, power supply control, etc.). The reliability offered by the TETRA network and the autonomy provided by a high-capacity power bank makes the automatic test kit a proper solution (primary or secondary) for a redundant monitoring architecture.

This benchmarking solution opens the road for future research in the form of a compact portable system supplemented with location information that can be used for mobile georeferenced measurements of network coverage and automated service testing. For example, automated testing of voice quality can be integrated in this benchmarking solution by implementing a test procedure similar to perceptual evaluation of speech quality (PESQ), which is a result of ITU-T efforts to standardize the end-to-end speech quality of narrow-band telephone networks and speech codec [37].

### 8.5. Monitoring and Control

**Figure 11:**
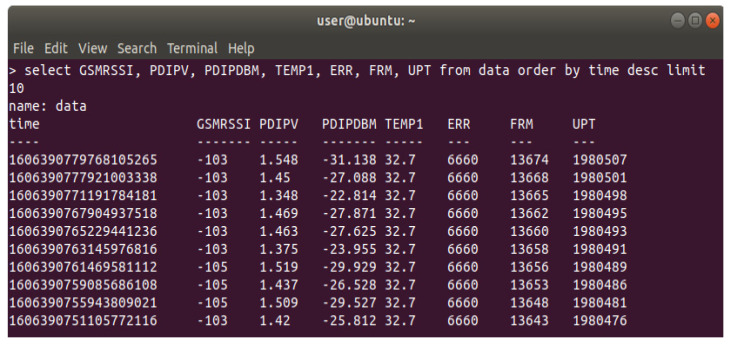
A sample of values stored in database, both ADC reading and converted values.

The testing and validation stages for monitoring and controlling technical solutions were performed in order to identify the availability thresholds and performances of our optical repeater. First method was to obtain the correct values that are logged in the database. These values were obtained from sampling the analogue signals provided by the sensors and converting the voltage values provided by board’s ADC to corresponding units (e.g., temperature °C, power dBm, etc.), either by a linear formula or by the means of a lookup table, for non-linear conversions. Queried results from database are shown in Figure 11:

- GSM RSSI—received signal strength indicator for local GSM service;
- PDIPV—power detector, ADC reading (volts);
- PDIPDMB—power detector, measured power (dBm);
- TEMP1—power amplifier, measured temperature (°C);
- ERR—control board, error counter;
- FRM—control board, data frame counter;
- UPT—control board, uptime.

In order to monitor the functional stability and the performance of the system, we considered three parameters (ERR, FRM, UPT), which can be used as quality indicators for error control mechanism (e.g., number of errors over uptime or over the number of data frames). 

**Figure 12:**
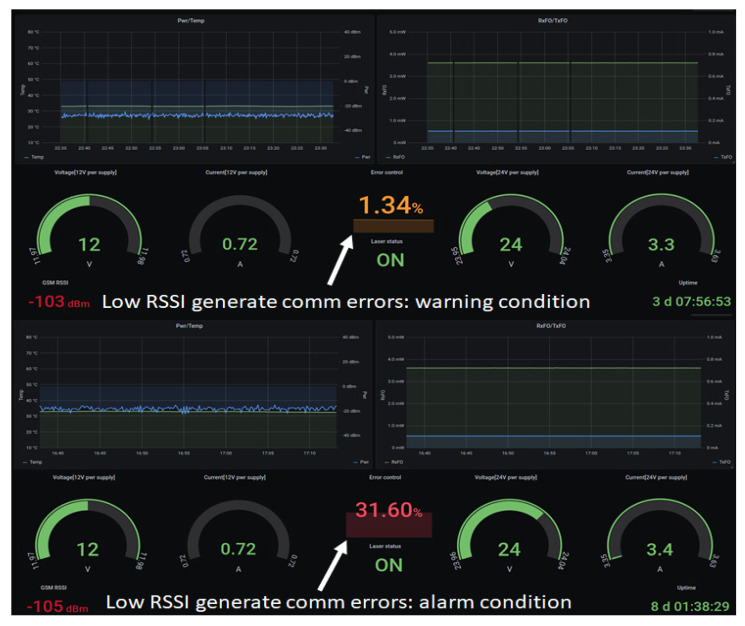
Error control with warning and alarm indication.

Validation tests were performed in areas with low GSM RSSI, where the number of communication errors increased dramatically, from a warning state (e.g., 1.34%) to an alarm state (e.g., 31.6%) considering the number of errors in relation to uptime, as shown in Figure 12. Being designed as a connection-oriented monitoring solution, the dashboard reports low GSM RSSI measured by the GSM modem which correlates with the error control parameter indicating a communication issue that may impact accessing the status information and limit the control function. Although a percentage of 1.34% communication errors can be accepted considering a low RSSI of −103 dBm, a decrease of only 2 dB to −105 dBm definitely is not acceptable, as the error control increases to a value of 31.6%, indicating that the repeater is close to being isolated from the monitoring network, as indicated in Figure 12. Please note that TETRA services were not influenced by low SNR conditions for GSM communication, but only the monitoring and control solution. In such cases, redundancy, for example, a supplementary connection over ethernet, should be implemented as a second communication link for monitoring and controlling continuity assurance. The separation between services and control media is an important aspect that should be taken into consideration from the design stage, as it should not represent a single point of failure. For example, if the communication link for control and monitoring solutions is lost, services should not be affected. The same statement applies if the service is lost: the control and monitoring applications should be able to indicate an alarm.

An important element to mention is that testing and validation was performed in a bidirectional way:

- Network to mobile stations: RF coverage distribution, link budget calculation, gain considerations, monitored parameters, control mechanism and service-side testing;
- Mobile stations to network: service-side testing using the automated test kit for SDS service and voice services tests;
- The reason for this approach was to gain confidence in the solution by correlating the information provided by our monitoring and control solution with an independent one (e.g., a system that measures the RSSI and counts sent messages).

### 8.6. Quality Measurements

**Figure 13:**
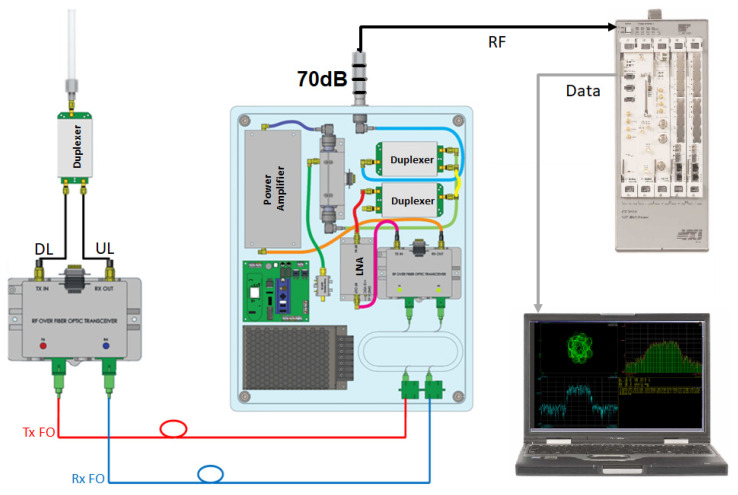
Modulation quality measurement testbed.

This evaluation was performed using an Agilent (Keysight) Vector Signal Analyzer in order to determine the influence of the active stages over the digital modulation ($\frac{\pi }{4}DQPSK$). The testbed for quality measurements is presented in Figure 13.

**Figure 14:**
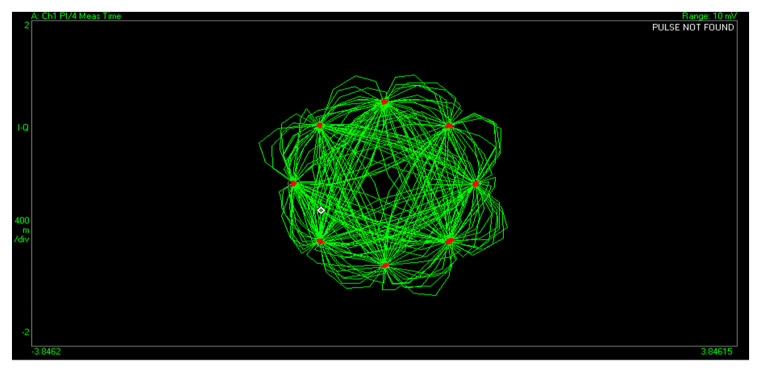
The constellation corresponding to *π* over 4 DQPSK modulation.

In order to perform the modulation quality check, one can observe on the IQ diagram a feature of the vector signal analyzer, as shown in Figure 14. The IQ diagram is a representation of IQ baseband signal in time domain as transitioning from one symbol to another.

The $\frac{\pi }{4}DQPSK$ modulation used in TETRA encodes the data bits in the phase change between consecutive symbols, resulting in eight symbol positions and two bits per symbol, as shown in Table 2 and Figure 14:

**Table 2:** Bit values related to phase change.

Id	Bit Values	Phase Change
1.	00	$\frac{\pi }{4}$
2.	01	$\frac{3\pi }{4}$
3.	10	$-\frac{\pi }{4}$
4.	11	$\frac{3\pi }{4}$

**Figure 15:**
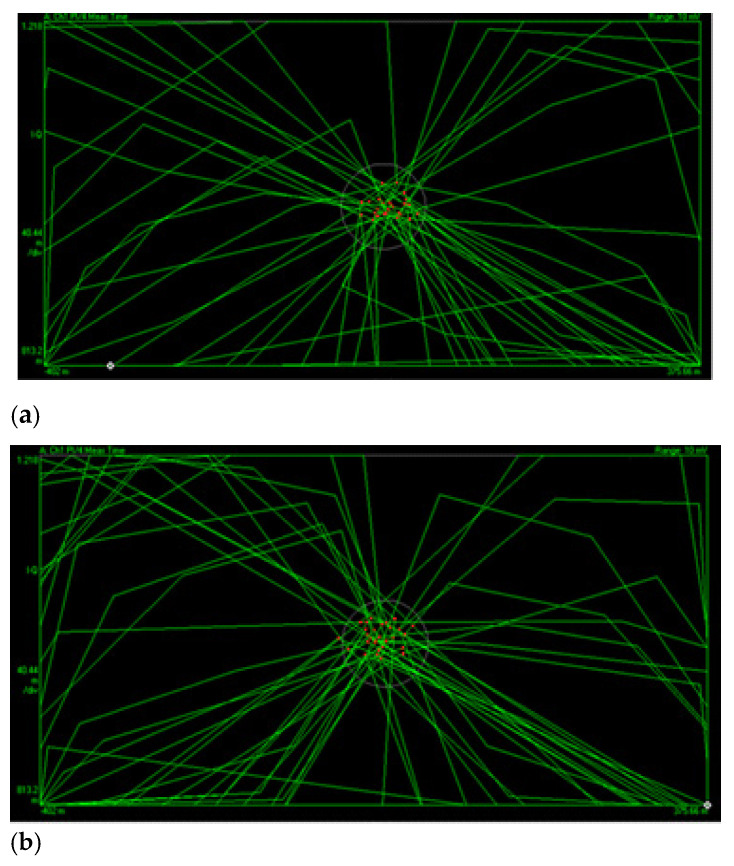
Detailed visualization of symbol states with respect to the error tolerance (circle): (**a**) symbol distribution for an EVM of 1.84%, (**b**) symbol distribution for an EVM of 2.91%.

In an ideal transmission, all symbols corresponding to each ideal state in the diagram should overlap. The real scenario, though, will produce a dispersion around the ideal points. The vector signal analyzer implements the quality limits imposed by the standards [1,2,3] by defining an error circle around each ideal point. The distribution of detected symbols with respect to the error circle offered a fast and comprehensive indication regarding the quality of the service provided by our optical repeater, as shown in Figure 14 and Figure 15.

The first conclusion reached is that the symbol distribution is grouped inside the reference circle, with no visible influence from interferences, phase noise or added noise, as shown in Figure 14.

The next quality evaluation was to perform multiple measurements over error vector magnitude (EVM) and logging the worst and the best results, according to Figure 15 and Table 3.

EVM and spectrum measurements represent good practice, not only in the laboratory but also in a real operational environment, as they may reveal equipment malfunction or electromagnetic interferences that may produce an interruption of services.

The parameters presented in Table 3 represent an in-depth assessment regarding the signal quality and are defined as follows [38,39]:

- *EVM—*obtained by determining the error vector which represents the difference vector from the ideal and measured symbol positions. The higher the EVM, the greater the displacement from the ideal state, as shown in Figure 15.
- *MagErr—*The IQ magnitude error represents the difference in amplitude between the ideal and the measured state.
- *PhaseErr—*The IQ phase error represents the phase difference between the ideal and the measured state with respect to detected symbols.
- *FreqErr—*The error of the instantaneous frequency of the measured signal compared to the reference, relative to the detected symbols.
- *IQ Offset—*Reflects the deviation of the real IQ diagram from the reference. The IQ offset is generally produced by the carrier feedthrough introducing a DC offset to I and Q data. A high IQ offset may affect the receiver’s ability to correctly detect the symbol.
- *Quad Error—*a measure of distortion and reflects the deviation from the ideal 90 degrees between I and Q components.
- *Amplitude droop—*Represents the power decrease of the transmitted signal relative to the symbol.
- *Gain imbalance—*Evaluated in a predefined measurement interval, the gain imbalance is determined as a logarithmic ratio between the magnitudes of the I and Q components.

With the worst EVM values of 2.91% and best 1.84% (5% being considered a quality threshold) according to [1], we concluded that the modulation quality is not affected by our design implementation. The conclusion was also validated by IQ diagram analysis and practical tests performed as voice calls and SDS messaging.

## 9. Results and Discussion

Designing an optical repeater, conducting tests and integrating the results in an experimental network is a real challenge when one aims for performance, reliability, availability and scalability at the same time. Either we deploy a single optical repeater or several repeaters that form a cluster or even an entire network, the technical solution should be integrated seamlessly in corresponding radio communication systems, according to purpose.

Various deployment scenarios and pick-up modes described in Section 3 and Section 4 offer the benefit of flexibility in installation and operation. For example, in each deployment scenario there are always two pick-up modes available: by antenna or via direct connection to the base station or another repeater. While the former mode can be performed at any site with the disadvantage of receiving a low RF signal depending on the distance to the base station/repeater, the latter is not signal restrictive, but the pick-up system should be installed in the same site. On the optical repeater side, the gain was determined for both paths, resulting in 52.6 dB for DL and 65.6 dB for UL considering the 20 dB gain offered by the pick-up system on the UL path.

Link budget evaluation was performed, as described in Section 6, and a radiating cable (leaky feeder) installation scenario was considered. The results indicated that a length of up to 3.71 km of radiating cable is feasible for a proper communication.

The monitoring of the state of the optical repeaters and function control are described in Section 7 and shown in Figure 4 and Figure 5 for normal operation conditions. In order to validate the solution, a series of tests were executed, as described in Section 8. A 2 km length, radiating-cable scenario was simulated, as described in Section 8.1 and Figure 6. The test was successful, as we could initiate and receive calls on our mobile station. The sensitivity threshold of a pair of RF over OF converters with respect to TETRA services was determined, as described in Section 8.2. The result was that we could exceed the 15 dBo limit provided by the manufacturer by 8 dBo without degrading the TETRA services. We concluded that an extension by one optical repeater could be possible with the cost of reduced RF coverage. However, in an operational scenario, care should be taken in designing the system due to the fact that, besides the attenuation, another limitation is introduced by chromatic dispersion which produces different propagation speeds for different wavelengths. Limiting the transmission distance, chromatic dispersion represents the differential delay (measured in ps) for a wavelength with a width of 1 ns that propagates through a fiber of 1 km length. For a conventional fiber (e.g., G.652D) [40,41] the chromatic dispersion coefficient for a 1310 nm wavelength is negligible but for 1550 nm is 17 ps/(nm×km). High speed transmissions, especially those using dense wavelength division multiplexing technology, are the most affected by chromatic dispersion and need compensation. For example, a transmission of 10 Gbps on a G.652D fiber at a wavelength of 1550 nm will be limited at 61 km, while an upgrade to 40 Gbps will reduce the distance below 4 km without compensation measures.

As a measure of usability, a service-side test validated the solution with 98% success, as described in Section 8.4. Several improvement possibilities can be explored.

Availability and performance tests for the communication link were executed and results were described in Section 8.5. Low RSSI for mobile data connection can produce a warning state or an alarm state. This limitation was considered starting with the design phase by using a redundant link as a second communication solution.

Quality was tested by voice calls, messages and by measuring the modulation parameters as described in Section 8.6. The results confirmed that the prototype does not degrade the quality.

Besides the benefits and advantages brought by the proposed solution, there are also some drawbacks and limitations generally found in repeater deployment solutions. For example, although an extension of the radio coverage is obtained, the capacity is not improved as the serving base station will have to manage an increased number of users. A solution to this issue could be repeating more than one base station. Another limitation is enforced by the daisy-chain topology which limits the number of optical repeaters due to the optical splitters needed in the chain. For example, a four-repeater chain will need three optical splitters that, if carefully chosen (in sequence of unbalanced balanced splitters), will introduce a minimum 6.4 dBo supplementary attenuation on the optical link (12.8 dB equivalent RF attenuation). Another drawback example can be considered in the use of two optical fibers, one for the downlink and another for the uplink path. This choice can be considered resource-intensive in deployments where fiber availability is an issue. An alternative might be to multiplex on a single fiber both the downlink and uplink using devices such as optical add-drop multiplexers, filters, directional couplers or optical circulators and evaluating the effect that any supplementary attenuation introduced by these elements may have on radio coverage and TETRA services. The proposed solution offers this possibility by externally coupling these optical elements at each repeater site.

## 10. Conclusions

The subject of this project represents a technical challenge as it is meant to provide large-scale radio coverage over various topologies in a distributed functionality. 

However, optical repeater design for critical communications is a proper technical solution that provides extended radio frequency coverage considering various numbers of operational requirements. It also can sustain all the aforementioned scenarios related to coverage requirements, signal pick-up, selectivity and applications. Besides functional testing and evaluation, the service quality is targeted, as it is a key aspect in user acceptance. Any service degradation should be prompted by the monitoring and control application that also has a mechanism of root cause analysis. For example, in the cluster architecture presented in Figure 1, where optical repeaters are installed in different geographic locations, the optical link is monitored in a bidirectional manner: laser power received from the neighbor optical repeater (RxFO) and the laser bias current (TxFO) to the next optical repeater in the chain. Monitoring both parameters allow the identification of the faulty source. Another example is the output RF power parameter evaluation. A low value may indicate a local power amplifier fault, but if the neighbor optical repeater also indicates low values than the root cause analysis may lead to the pick-up system and further, to the base station.

The monitoring and control applications can be operated in a centralized and hierarchized way, starting from component, repeater, cluster and ending with an entire network of repeaters. The flexibility of deployment is one of the most important characteristics as it allows a cost-effective solution to be planned and deployed, starting from a simple point-to-point topology and ending with a multi-clustered network. Another important element is the pick-up mode selection as it offers various solutions to classic issues such as: costs of deployment, low pick-up power level, long distance to service areas, coverage in the pick-up site. An innovative characteristic besides the flexibility of the deployment can be considered the ability to perform signal pick-up and TETRA coverage in the same site at a low cost and optical link attenuation. Another novel idea is the testing and validation automation that can be extended to various types of applications. 

Mission-critical services are operated in a seamless manner having the same prioritization and security measures provided by the network. Allowing a versatile installation and assuring extended coverage for different scenarios, the solution proposed in this paper improves the operational mobility and service usability responding to various mission-critical objectives. 

Future work consists of implementing redundancy at service level in order to obtain high availability and developing optical repeater solutions for mission-critical communications on other wireless networks, based on the same principles and know-how.

## Figures and Tables

**Table 1:** Gain calculation.

Gain [dB]	Components Gain/Attenuation [dB]							
	FO_loss_	FO_conv_	PA	LNA	DC	DUP	Cable	Total
G_DL_	−7.4 *	20	46	-	−1	−2	−3	G_D__L_ = 52.6 dB
G_UL_	−7.4 *	20	-	30	-	−4	−3	G_UL_ = 35.6 dB **

Note that 7.4 dB (*) RF loss is equivalent to 3.7 dBo optical loss, and balanced gain is obtained by using a supplementary LNA at the pick-up system, on the UL RF path (**). Overall gain for the UL path becomes **65.6 dB**, considering both optical repeater and pick-up system gain.

**Table 3:** EVM evaluation.

Parameter	Measured values	
	Val_Min_	Val_Max_
EVM	1.84%	2.91%
MagErr	1.18%	1.89%
PhaseErr	0.805°	1.268°
FreqErr	−158.88 Hz	−114.45 Hz
IQ Offset	−35.48 dB	−34.859 dB
Quad Error	0.456°	0.554°
Amplitude Droop	177.5 µdB/sym	178.2 µdB/sym
Gain Imbalance	−0.092 dB	−0.089 dB

## Data Availability

Not applicable.

## References

[1] European Standard, Terrestrial Trunked Radio (TETRA), Voice+Data (V+D), Part 2: Air Interface, (AI), (2016-08).

[2] Technical Specification, Terrestrial Trunked Radio (TETRA) Voice+Data (V+D) Part 2: Air Interface (AI), (2019-01).

[3] European Standard, Terrestrial Trunked Radio (TETRA); Voice plus Data (V+D); Part 1: General Network Design (2020-04).

[4] Abdulrahman Y. (2020). Public Safety Networks from TETRA to Commercial Cellular Networks. Public Safety Networks from LTE to 5G.

[5] Natasha F., Usman U.K., Satria R. (2020). Analysis of Interference between LTE System and TETRA System in the 800 MHz Band. Aviat. Electron. Inf. Technol. Telecommun. Electr. Control..

[6] Velianitis G., Adel K., Kotrba S., Manavalan B.P. (2018). Comparison of VoIP and TETRA Regarding Security in a Safety Critical Environment. J. Comput..

[7] Umlauft M., Raffelsberger C. Evaluation of TETRA SDS Performance and GPS Accuracy in Real-World Scenarios. Proceedings of the 2019 15th International Conference on Telecommunications (ConTEL) IEEE.

[8] Höyhtyä M., Lähetkangas K., Suomalainen J., Hoppari M., Kujanpää K., Ngo K.T., Kippola T., Heikkilä M., Posti H., Mäki J. (2018). Critical communications over mobile operators’ networks: 5G use cases enabled by licensed spectrum sharing, network slicing and QoS control. IEEE Access.

[9] Grigoreva E., Shrivastava D., Machuca C.M., Kellerer W., Dittrich J., Wilk H., Zimmermann H.M. Heterogeneous wireless access network protection for ultra-reliable communications. Proceedings of the IEEE Vehicular Networking Conference (VNC).

[10] Kaleem Z., Yousaf M., Qamar A., Ahmad A., Duong T.Q., Choi W., Jamalipour A. (2019). UAV-empowered disaster-resilient edge architecture for delay-sensitive communication. IEEE Netw..

[11] Dunlop J., Girma D., Irvine J. (2013). Digital Mobile Communications and the TETRA System.

[12] Ulema M. (2019). Fundamentals of Public Safety Networks and Critical Communications Systems: Technologies, Deployment, and Management.

[13] Lähdekorpi P., Isotalo T., Khan S.U., Lempiäinen J. Implementation aspects of RF-repeaters in cellular networks. Proceedings of the 21st Annual IEEE International Symposium on Personal, Indoor and Mobile Radio Communications.

[14] Grecu I.V., Mădălina-Varvara B.A., Aloman A., Petrescu N.V., Grecu E.A. Main Solutions for Extending Radio Coverage in TETRA Networks. Proceedings of the 2018 10th International Conference on Electronics, Computers and Artificial Intelligence (ECAI).

[15] Harb N., Valderrama C., Pisane J. FPGA-based digital tunable wireless transceiver for the TETRA-TETRAPOL bands. Proceedings of the 2017 12th IEEE International Symposium on Industrial Embedded Systems (SIES).

[16] Emin B., Bașbug S. Digital Repeater Design for Single Chip Radio Transceiver. Proceedings of the International Turkic World Congress on Science and Engineering, UTFEM 2019.

[17] Rolain Y., Van Moer W., Pintelon R., Schoukens J. (2006). Experimental characterization of the nonlinear behavior of RF amplifiers. IEEE Trans. Microw. Theory Tech..

[18] RFoverFiber. www.opticalzonu.com.

[19] Axiotis D.I., Salkintzis A.K. (2010). Packet data messaging over TETRA: Network performance analysis. Wirel. Netw..

[20] Lim C., Tian Y., Ranaweera C., Nirmalathas T.A., Wong E., Lee K.L. (2019). Evolution of radio-over-fiber technology. J. Lightwave Technol..

[21] Hadi M.U., Hyun J., Salman G., Traverso P.A., Tartarini G. (2019). Optimized digital radio over fiber system for medium range communication. Opt. Commun..

[22] Ali A.H., Farhood A.D., Maham K.N. (2020). Analysis of a framework implementation of the transceiver performances for integrating optical technologies and wireless LAN based on OFDM-RoF. Int. J. Electr. Comput. Eng..

[23] del Rey Carrión D., Juan-Llácer L., Rodríguez J.V. (2019). Radio planning considerations in tetra to lte migration for ppdr systems: A radioelectric coverage case study. Appl. Sci..

[24] Products. www.vialite.com.

[25] Nassery A., Ozev S., Verhelst M., Slamani M. Extraction of EVM from transmitter system parameters. Proceedings of the 2011 Sixteenth IEEE European Test Symposium.

[26] Kumar P., Sharma S.K., Singla S., Gupta V., Sharma A. (2021). A review on mmWave based energy efficient RoF system for next generation mobile communication and broadband systems. J. Opt. Commun..

[27] Chochliouros I.P., Spiliopoulou A.S., Lazaridis P.I., Zaharis Z.D., Spada M.R., Pérez-Romero J., Blanco B., Khalife H., Khaleghi E.E., Kourtis M.A. (2021). 5G for the Support of Public Safety Services. Wirel. Pers. Commun..

[28] Trial Guidance Methodology. www.drive-project/trial-guidance-methodology.

[29] Products, Amplifiers. www.minicircuits.com.

[30] Products. www.amphenolprocom.com.

[31] Agilent PN 89400-14, Product Note, Using Error Vector Magnitude Measurements to Analyze and Troubleshoot Vec-tor-Modulated Signals. www.keysight.com.

[32] Wireless Off-Air Repeaters for Two-Way Radio Systems. www.celab.se.

[33] Wireless Fibre-DAS Solutions for Two-Way Radio. www.celab.se.

[34] Stockholm Metro Project-Case Study: Innovation Making TETRA Indispensable. www.axellwireless.com/app/uploads/2021/01.

[35] Products. www.axellwireless.com/solutions/tunnels/.

[36] Products, TETRA. www.bhe-mw.eu/products/tetra.

[37] (2018). Perceptual Evaluation of Speech Quality (PESQ), ITU-T Recommendation P.862. https://www.itu.int/rec/T-REC-P.862.

[38] (2004). Vector Signal Analysis Basics, Agilent, Application Note 150-15. https://www.comtest.ro/biblioteca-tehnica/5989-1121EN-AN-150-15-VSA-Basics.pdf.

[39] (2021). R&S®FPS-K70 User Manual. https://www.rohde-schwarz.com/uk/manual/r-s-fps-k70-user-manual-manuals-gb1_78701-54617.html.

[40] (2009). Transmission Systems and Media, Digital Systems and Networks, Characteristics of a Non-Zero Dispersion-Shifted Single-Mode Optical Fibre and Cable.

[41] (2009). Transmission Systems and Media, Digital Systems and Networks, Characteristics of a Single-Mode Optical Fibre and Cable.

